# Cyclic pulsation stress promotes bone formation of tissue engineered laminae through the F-actin/YAP-1/β-Catenin signaling axis

**DOI:** 10.1038/s41536-021-00164-w

**Published:** 2021-09-06

**Authors:** Linli Li, Hailong Li, Yiqun He, Han Tang, Jian Dong, Xujun Chen, Feizhou Lyu, Youhai Dong

**Affiliations:** 1 Department of Orthopedics, Shanghai Fifth People’s Hospital, Fudan University, Shanghai, China; 2 Department of Orthopedics, Huashan Hospital, Fudan University, Shanghai, China

**Keywords:** Mesenchymal stem cells, Checkpoint signalling, Stem-cell biotechnology

## Abstract

Mechanical loads are fundamental regulators of bone formation and remodeling. However, the molecular regulation of mechanotransduction during vertebral laminae regeneration remains poorly understood. Here, we found that cerebrospinal fluid pulsation (CSFP) stress—cyclic pulsation stress—could promote the osteogenic and angiogenic abilities of rat mesenchymal stromal cells (MSC), thereby promoting tissue-engineered laminae’s bone and blood vessel formation. In the process, F-actin relayed CSFP stress to promote the nuclear translocation of YAP1, which then decreased the degradation and promoted the nuclear translocation of β-Catenin. In turn, the nuclear translocation of β-Catenin promoted the osteogenic differentiation and angiogenic abilities of MSC, thereby promoting tissue-engineered laminae’s bone and blood vessel formation. Thus, we conclude that CSFP promotes the osteogenesis and angiogenesis of tissue-engineered laminae through the F-actin/YAP-1/β-Catenin signaling axis. This study advances our understanding of vertebral laminae regeneration and provides potential therapeutic approaches for spinal degeneration after spinal laminectomy.

## Introduction

Mechanical forces are part of environmental cues that are sensed and responded to during bone development and regeneration^1,2^. Apart from the complex networks of biochemical signaling that direct the differentiation during bone formation, bone cells can also constantly respond to mechanical cues such as hydraulic pressure, fluid shear stress, pulsation stress, or substrate stiffness from the microenvironment^2–5^. Mechanotransduction, the conversion of mechanical forces into biological signals, is a fundamental physiologic process critical for bone formation^6,7^. Our previous studies have shown that cerebrospinal fluid pulsation (CSFP) stress, as cyclic pulsation stress, could promote the osteogenesis, vascularization, and remodeling of tissue-engineered laminae (TEL)^8–10^. However, the underlying mechanotransduction and osteogenic process that regulates the vertebral laminae regeneration remains poorly understood.

In the process of mechanotransduction, cells sense and counterbalance the extracellular forces by adjusting their own tensional state through actomyosin contractility and organization of the F-actin cytoskeleton^11^. Then the mechanical instructions are executed in the nucleus through specific transcription factors. Recent discoveries found that YAP and TAZ, two highly related transcriptional regulators, could convert various mechanical inputs into transcriptional responses and coherent biological functions^12^. TRPV4 mediates fluid shear stress-induced calcium signaling and early osteogenic differentiation in bone mesenchymal stem cells^13^. Piezo1/2 can also relay fluid shear stress to activate Ca^2+^ influx to promote concerted activation of NFATc1, YAP1, and β-Catenin transcription factors by inducing their dephosphorylation as well as NFAT/YAP1/β-Catenin complex formation^5,14^.

Wnt/β-Catenin signaling pathway played a crucial role in skeletal development and bone regeneration, which functions through the accumulation and translocation of β-Catenin into the nucleus^15^. The nucleus translocation of β-Catenin leads to activation of TCF/LEF, a set of transcriptional activators, which will trigger a transcriptional response^16^. The activity of cytoplasmic β-Catenin is regulated by a β-Catenin destruction complex comprising Axin, APC, and GSK3. The destruction complex is also a critical checkpoint coordinating both β-Catenin and YAP/TAZ activity^17^. Cytoplasmic YAP/TAZ favors the recruitment of β-transducin repeats-containing protein (β-TRCP) to the destruction complex and facilitates β-Catenin degradation^18–20^. Therefore, YAP/TAZ nuclear translocation can lead to β-Catenin nuclear localization. Zhou et al.^5^ also proved that fluid shear stress could promote the concerted activation of NFAT/YAP1/β-Catenin complex and, thereby, bone formation.

Our present study investigated how CSFP regulated the osteogenic and angiogenic abilities of mesenchymal stromal cells (MSC) and bone formation of TEL during vertebral laminae reconstruction. We found that F-actin relayed cyclic pulsation stress to inhibit the degradation and promote nuclear translocation of β-Catenin and YAP1. The nuclear translocation of β-Catenin and YAP1 promoted the bone formation and vascularization of TEL. Our study advances the understanding of vertebral laminae regeneration and potential therapeutic approaches for spinal degeneration after spinal laminectomy.

## Results

### CSFP promoted the osteogenic and angiogenic abilities of rat MSC

**Fig. 1:**
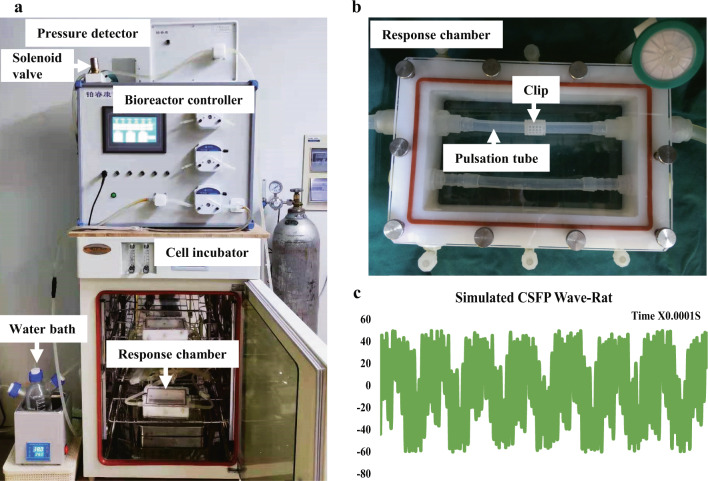
Cerebrospinal fluid pulsation bioreactor system. **a** The modules of the bioreactor system. **b** The response chamber of the bioreactor, the MSC-PLGA complexes were fixed on the pulsation tube using the clip. **c** The mimicked rat cerebrospinal fluid stress wave by the bioreactor system.

**Fig. 2:**
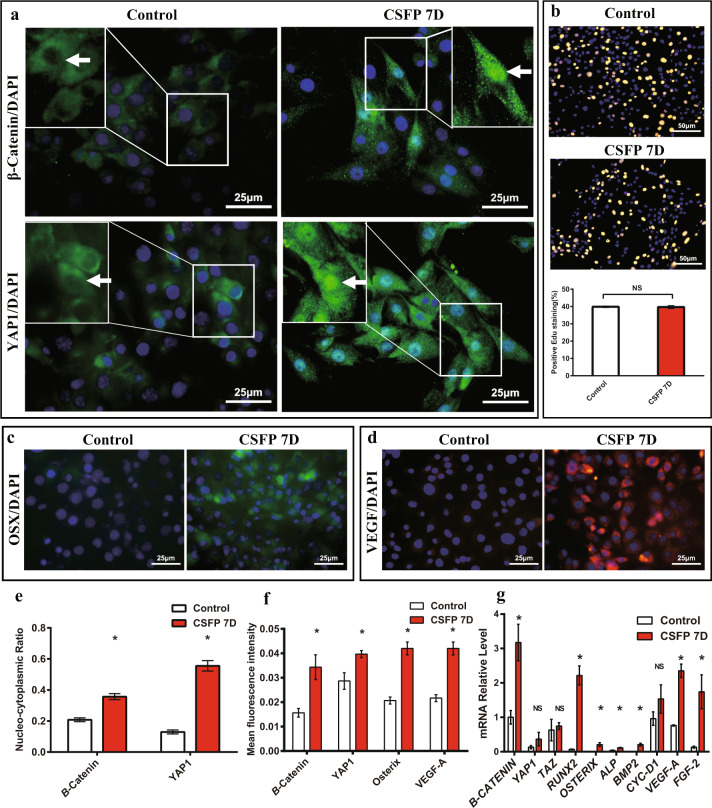
CSFP promoted the expression and nuclear translocation of β-Catenin and YAP1, and promoted the osteogenic and angiogenic abilities of MSC. **a** IF staining of β-Catenin and YAP1, and the white arrow shows the nucleus. **b** Edu staining showed no statistical difference between two groups. IF staining of Osterix (**c**) and VEGF-A (**d**). **e** The nucleo-cytoplasmic ratio of β-Catenin and YAP1 in the CSFP 7D group was statistically higher than that of the Control group. **f** The mean fluorescence intensity of β-Catenin, YAP1, Osterix, and VEGF-A in the CSFP 7D group was statistically higher than that of the Control group. **g** QRT-PCR analysis. Data are presented as mean ± SEM, and ^*^P < 0.05.

To examine whether CSFP could regulate the proliferative, osteogenic, or angiogenic abilities of rat MSC derived from Wharton’s Jelly, we measured the CSFP stress of rats at the lumbar vertebrate by Codman cerebrospinal pressure detector (Johnson & Johnson, NJ, USA), and found that the amplitude of the CSFP stress at the lumbar level was about 100 Pa with the frequency of 5 Hz (Jian Dong and Linli Li, 2018, Unpublished data). We utilized the bioreactor (Fig. 1a, b) to mimic the cyclic cerebrospinal pulsation stress wave of rats at the lumbar level with the pulsation amplitude of 100 Pa and frequency of 5Hz (Fig. 1c). The experiment was divided into the Control group (without CSFP intervention) and the CSFP 7D group (with CSFP intervention for 7 days). In both groups, the percentage of MSC in the DNA synthesis phase (S phase) is about 40% and showed no difference; the mRNA expression levels of *CYC-D1* also showed no difference (Fig. 2b, g). CSFP could promote the expression and nuclear translocation of β-Catenin (Fig. 2a, e, f), and the mRNA expression levels of *β-CATENIN* in the CSFP7D group were also statistically higher than that of the Control group (Fig. 2g). CSFP had no significant effect on the mRNA expression of *YAP1* and *TAZ* on day 7 (Fig. 2g), but CSFP could promote the expression and nuclear translocation of YAP1, indicating that CSFP might inhibit the degradation of the cytoplasmic YAP1 (Fig. 2a, e, f). The expression levels of osteogenic genes such as *RUNX2, OSTERIX, ALP*, and *BMP2* in the CSFP 7D group were statistically higher than those of the Control group, confirmed by the IF staining of Osterix (Fig. 2c, f, g). CSFP also promoted the expression levels of angiogenic genes such as VEGF-A and FGF2 (Fig. 2d, f, g). These results suggested that CSFP could promote the expression and nuclear translocation of β-Catenin and YAP1, promote their osteogenic and angiogenic abilities, but have no effect on their proliferative abilities.

### CSFP promoted the osteogenic and angiogenic abilities through the regulation of β-Catenin

**Fig. 3:**
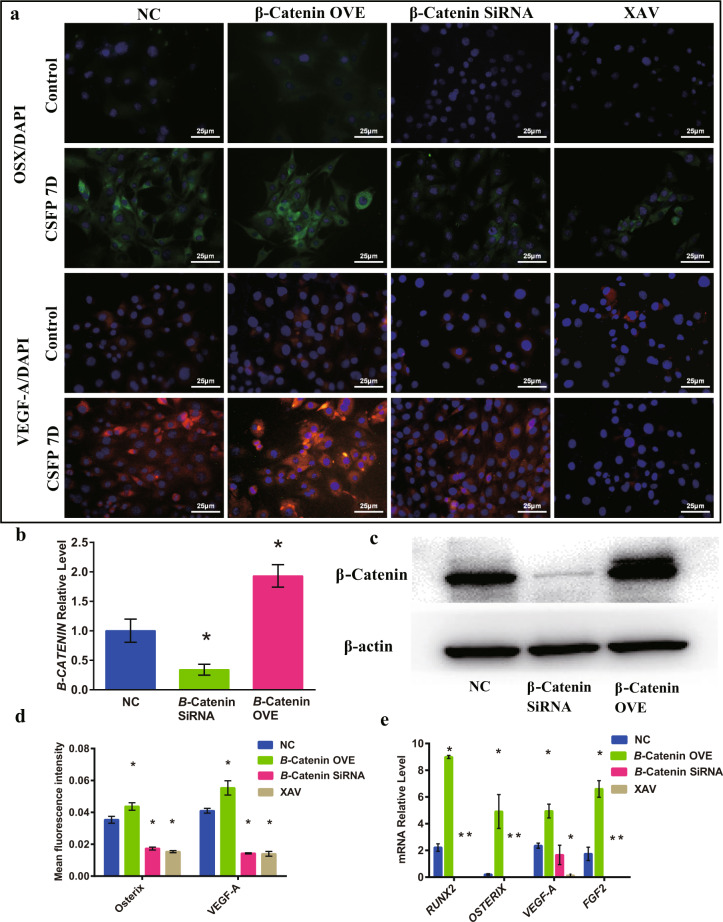
CSFP promoted the osteogenic and angiogenic abilities of MSC through the regulation of β-Catenin. **a** IF staining of Osterix and VEGF-A. **b** *β-CATENIN* mRNA expression levels in the normal control (NC), β-Catenin-knockdown (β-Catenin siRNA), and β-Catenin-overexpression (β-Catenin OVE) cell models. **c** β-Catenin protein expression levels in the NC, β-Catenin siRNA, and β-Catenin OVE cell models. **d** The mean fluorescence intensity of Osterix and VEGF-A in the β-Catenin OVE group was statistically higher than that of the NC group. Conversely, those in the β-Catenin siRNA and β-Catenin-inhibition (XAV) groups were statistically lower than that of the NC group. **e** QRT-PCR analysis. Data are presented as mean ± SEM, and ^*^P < 0.05 (experimental groups vs NC group).

We created a β-Catenin overexpression (β-Catenin OVE group) cell model by transfection of MSC with adenovirus vector overexpressing β-Catenin and created a β-Catenin-knockdown (β-Catenin siRNA group) MSC model by transfection of MSC with small interference RNA (siRNA) (Fig. 3b, c). We also created β-Catenin inhibition (XAV group) cell model by adding XAV-939 (a potent tankyrase inhibitor that targets Wnt/β-Catenin signaling by inhibiting tankyrase 1/2 and thereby stimulating β-Catenin degradation, 1.0 μM) to the culture medium. On day 7 with CSFP intervention, the β-Catenin OVE group showed increased mRNA expression levels of *RUNX2, OSTERIX, VEGF-A*, and *FGF2*, also confirmed by the IF staining of Osterix and VEGF-A (Fig. 3a, d, e). Conversely, the knockdown or inhibition of β-Catenin reduced the CSFP-promoted expression of osteogenic and angiogenic markers, inhibiting the osteogenic and angiogenic abilities of MSC (Fig. 3a, d, e). Edu staining showed that there was no statistical difference about the percentage of MSC in proliferating phase between the four groups (Supplementary Fig. 1). These results suggested that CSFP could promote the expression levels of osteogenic and angiogenic markers through the upregulation of β-Catenin.

### Inhibition of β-Catenin led to decreased bone and blood vessel formation

**Fig. 4:**
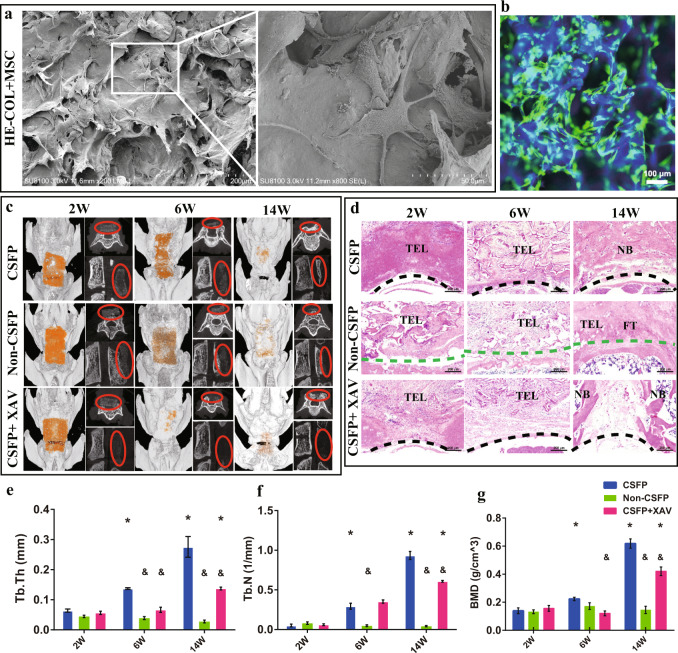
CSFP promoted the bone formation of tissue-engineered laminae through the regulation of β-Catenin. **a** SEM images of TEL. **b** Live/Dead staining of TEL. **c** Micro-CT scanning and 3-D reconstruction images at the 2nd, 6th, and 14th weeks, the pseudo-orange color shows the TEL, and the red dash circle shows the TEL or newborn bone. **d** HE staining of TEL at the 2nd, 6th, and 14th weeks. TEL: tissue-engineered laminae, NB: newborn bone, FT: fibrous tissue. **e** Trabecular thickness (Tb.Th). **f** Trabecular number (Tb.N). **g** Bone mineral density (BMD). Data are presented as mean ± SEM. ^*^P < 0.05 (comparison of the same group at different time points), ^&^*P* < 0.05 (comparison of different groups at the same time point).

In this part, we made CSFP rat models (CSFP group), CSFP isolation rat models (Non-CSFP group), and CSFP + β-Catenin inhibition rat model (CSFP + XAV group) (Supplementary Fig. 2) to examine whether the inhibition of β-Catenin could reduce bone and blood vessel formation. TEL were made by hydroxyapatite-collagen I scaffold and rat MSC. SEM examination showed the MSC formed finger-like filopodia and tightly adhered to the scaffold (Fig. 4a). Live/Dead staining also showed that almost all the MSC survived and adhered to the scaffold (Fig. 4b). The hydroxyapatite-collagen I scaffold exhibited excellent biocompatibility.

In the CSFP group, the newborn laminae gradually grew from bilateral vertebral pedicles to the middle and formed complete artificial laminae at the 14th week; for the Non-CSFP group, the TEL were gradually absorbed and almost no newborn bone formed; for the CSFP + XAV group, the bone formation rate was significantly lower than that of the CSFP group, and no complete artificial laminae were formed at the 14th week (Fig. 4c**)**. In the CSFP group, the scaffold degradation and trabecular bone formation proceeded orderly and alternately; for the Non-CSFP group, the scaffold degradation was dominant over the bone formation and almost no bone was formed; for the CSFP + XAV group, the scaffold degradation overrated bone formation, and there was still a lamina defect with the size of 400 μm at the 14th week (Fig. 4d). The number of trabecular bone (Tb. N), trabecular thickness (Tb. Th), and bone density (BMD) of the newborn laminae in the CSFP group were statistically higher than those of the Non-CSFP group and CSFP + XAV group at the 14th week (Fig. 4e–g).

**Fig. 5:**
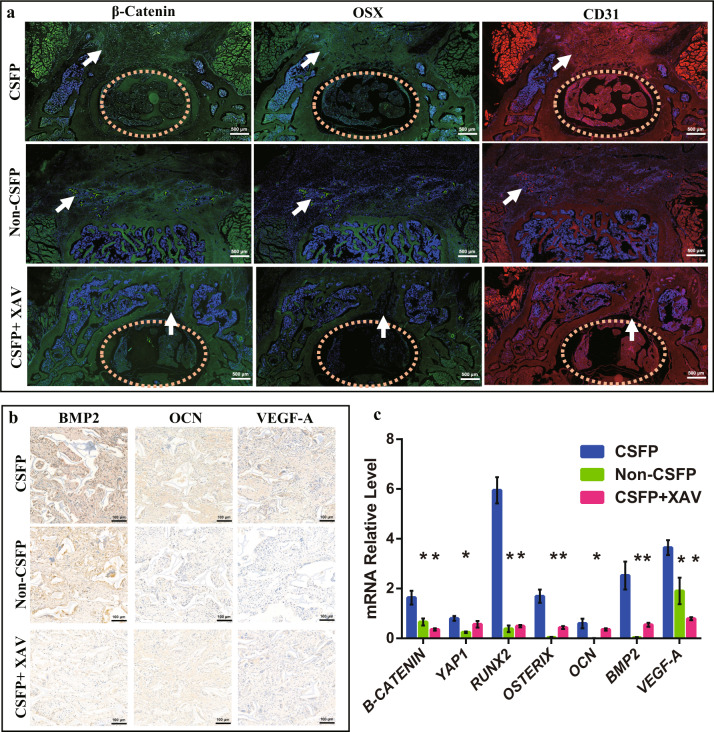
CSFP promoted the osteogenic and angiogenic abilities of tissue-engineered laminae through the regulation of β-Catenin. **a** β-Catenin, Osterix, and CD31 IF staining of tissue-engineered laminae (TEL) at the 14th week, the orange dash circle shows the vertebral canal, and the white arrow shows the TEL. **b** BMP2, OCN, and VEGF-A IHC staining of TEL at the 6th week. **c** QRT-PCR analysis at the 6th week. Data are presented as mean ± SEM, and ^*^P < 0.05.

**Fig. 6:**
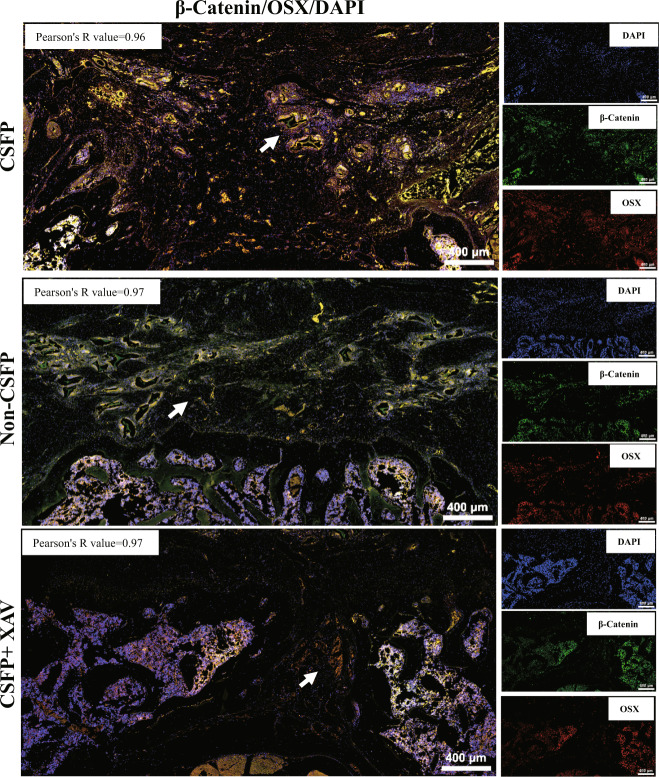
Double immunofluorescent staining revealed colocalization of β-Catenin (green) and Osterix (red). The white arrow shows the TEL.

The mRNA expression levels of *YAP1, β-CATENIN, RUNX2, OSTERIX, OCN, BMP2*, and *VEGF-A* in the CSFP group were statistically higher than those of the Non-CSFP group, confirmed by the IF staining of β-Catenin, Osterix, and CD31 and IHC staining of BMP2, OCN, and VEGF-A (Fig. 5a, b). Inhibition of β-Catenin could reduce the expression of *β-CATENIN, RUNX2, OSTERIX, BMP2*, and *VEGF-A*, but not that of *YAP1* (Fig. 5c). Double immunofluorescent staining revealed that the expression and localization trends of β-Catenin and Osterix were consistent, and the Pearson’s correlation value analyzed by ImageJ was about 0.97, indicating a strong positive correlation between β-Catenin and osteoblast differentiation (Fig. 6). These results suggested that CSFP could promote the expression levels of osteogenic and angiogenic markers, thereby promoting the bone and blood vessel formation of TEL through the regulation of β-Catenin.

### YAP1 knockdown inhibited the expression and nuclear translocation of β-Catenin and decreased the CSFP-induced osteogenic and angiogenic potential

**Fig. 7:**
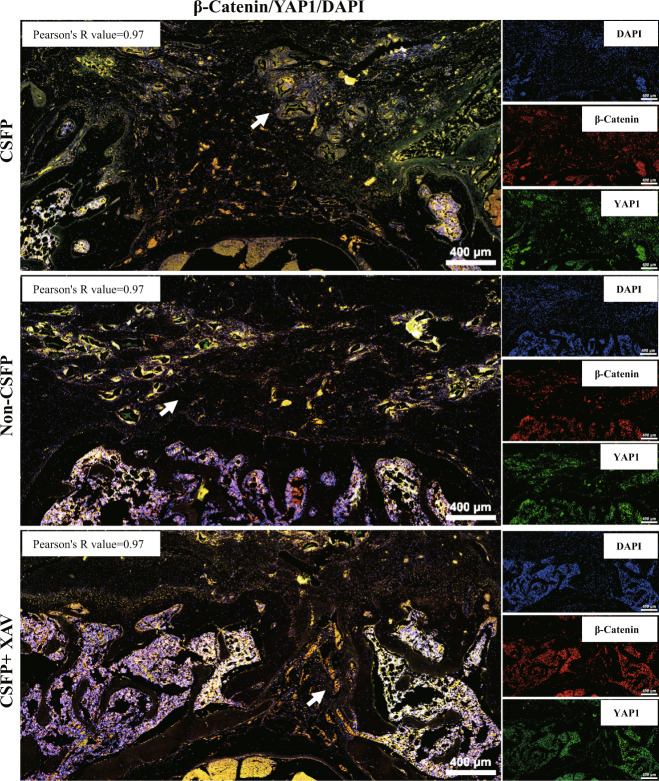
Double immunofluorescent staining revealed colocalization of β-Catenin (red) and YAP1 (green). The white arrow shows the TEL.

**Fig. 8:**
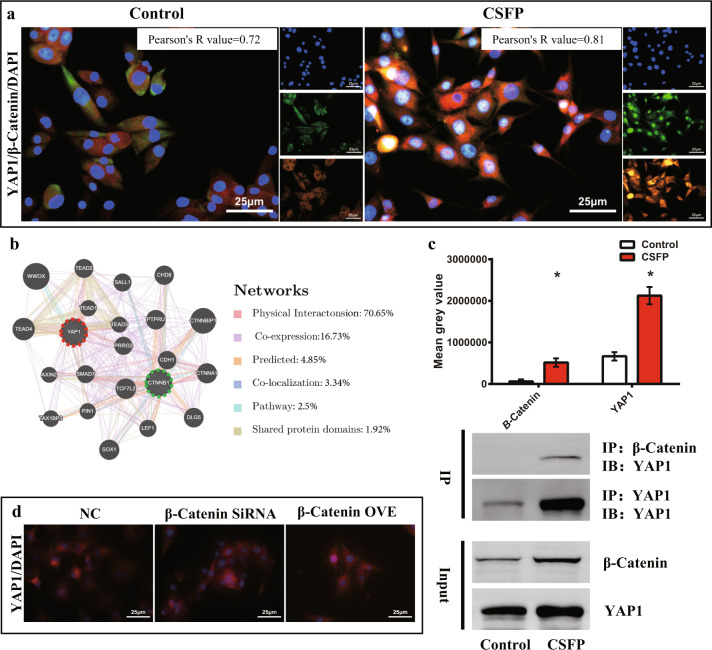
CSFP promoted the binding of β-Catenin and YAP1. **a** Double immunofluorescent staining revealed colocalization of β-Catenin (Red) and YAP1 (Green). **b** GeneMANIA database showed the probability of physical interactions between β-Catenin and YAP1 was 70.65%. **c** Co-IP analysis showed that CSFP promoted the binding of β-Catenin and YAP1. **d** Overexpression or knockdown of β-Catenin did not affect the activities of YAP1. Data are presented as mean ± SEM, and ^*^P < 0.05.

Double immunofluorescent staining revealed the coexpression and colocalization of β-Catenin and YAP1 in TEL with Pearson’s correlation of 0.97, indicating their strong positive correlation (Fig. 7). Then we found that the β-Catenin and YAP1 in the cells of control and CSFP groups also showed a coexpression and colocalization trend, and the Pearson’s correlation value was above 0.7, indicating a strong positive correlation (Fig. 8a). The GeneMANIA database (https://genemania.org/) predicted that the probability of protein–protein interactions between β-Catenin and YAP1 was about 70%, and the probability of their coexpression was about 17% (Fig. 8b). Then we founded that CSFP could promote the binding of β-Catenin and YAP1 by co-immunoprecipitation (Fig. 8c). These results showed a strong relationship between β-Catenin and YAP1. However, the overexpression or knockdown of β-Catenin could not regulate the expression or nuclear translocation of YAP1 (Fig. 8d).

**Fig. 9:**
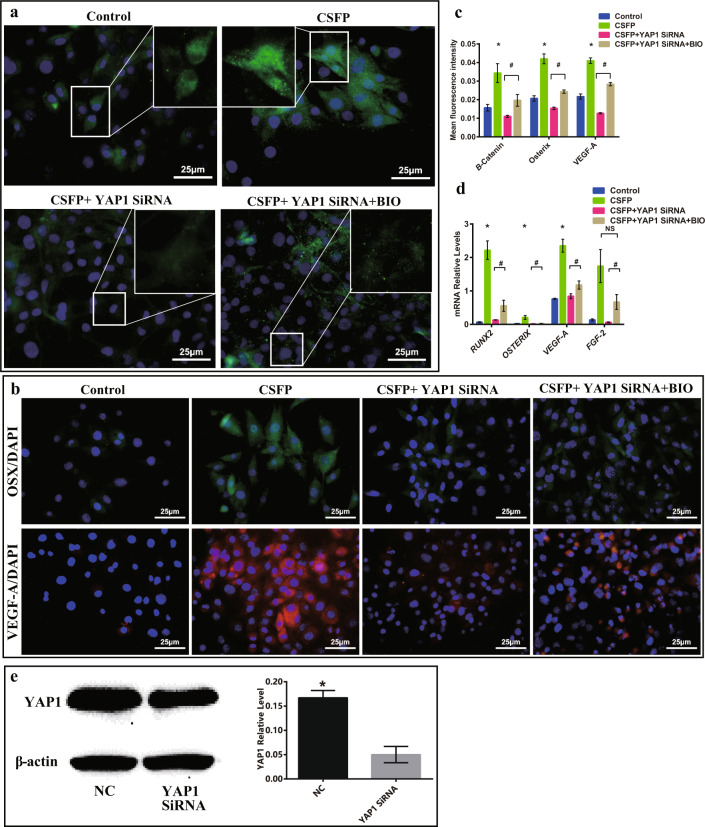
CSFP promoted the osteogenic and angiogenic abilities of MSC through the YAP1/β-Catenin axis. **a** IF staining revealed YAP-1 knockdown inhibited the expression and nuclear translocation of β-Catenin, and treatment with GSK-3β inhibitor recovered the β-Catenin expression. **b** IF staining revealed YAP-1 knockdown inhibited the expression of Osterix and VEGF-A, and treatment with BIO recovered their expressions. **c** The mean fluorescence intensity of β-Catenin, Osterix, and VEGF-A. **d** QRT-PCR analysis. **e** *YAP1* mRNA and YAP1 protein expression levels in the normal control (NC) and YAP1-knockdown (YAP1 siRNA) cell models. Data are presented as mean ± SEM, and ^*^P < 0.05. ^#^*P* < 0.05 (CSFP + YAP1 siRNA group vs CSFP + YAP1 siRNA + BIO group).

To investigate whether YAP1 could regulate the β-Catenin activity, we created the YAP1-knockdown (YAP1 siRNA group) MSC model by transfection of YAP1 siRNA into MSC (Fig. 9e). After 7 days of CSFP intervention, YAP1 knockdown reduced the expression levels of β-Catenin, Osterix, and VEGF-A and also reduced the nuclear translocation of β-Catenin (Fig. 9a–d). To test whether YAP1 regulates the osteogenic and angiogenic abilities of MSC through β-Catenin, we treated YAP1-knockdown MSC with BIO (2.0 μM), a potent specific inhibitor for GSK3 (a kinase that promotes β-Catenin degradation). Treatment with BIO reduced the degradation and promoted the nuclear translocation of β-Catenin in the CSFP + YAP1-knockdown MSC (Fig. 9a). In addition, the expression levels of Osterix and VEGF-A recovered significantly (Fig. 9b–d). These results showed that YAP1 mediated CSFP-induced osteogenic and angiogenic potential by regulating the degradation and nuclear translocation of β-Catenin.

### Disruption of F-actin polymerization inhibited nuclear translocation of YAP1 and β-Catenin

**Fig. 10:**
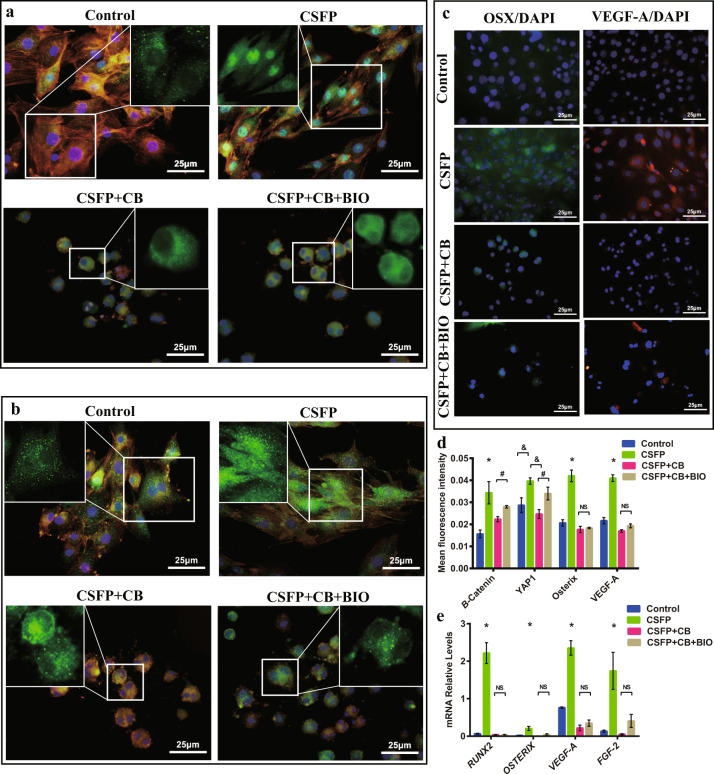
CSFP promoted the osteogenic and angiogenic abilities of MSC through the F-actin/YAP1/β-Catenin axis. IF staining revealed inhibition of F-actin polymerization, inhibited the nuclear translocation of β-Catenin (**a**) and YAP1 (**b**), and treatment with BIO did not recover their nuclear translocation. **c** IF staining of Osterix and VEGF-A. **d** The mean fluorescence intensity of Osterix and VEGF-A. **e** QRT-PCR analysis. Data are presented as mean ± SEM. ^*^P < 0.05, ^&^*P* < 0.05 (CSFP group vs Control or CSFP + CB group). ^#^*P* < 0.05 (CSFP + CB group vs CSFP + CB + BIO group).

To determine whether YAP1 and β-Catenin nuclear translocation is regulated by F-actin polymerization, we used Cytochalasin B (a cell-permeable mycotoxin binding to the barbed end of actin filaments and disrupting the formation of actin polymers, 20.0 μM, CB) to inhibit F-actin polymerization. Treatment of CB inhibited the cell elongation and CSFP-induced arrangement direction uniformity. The unclear translocation of β-Catenin and YAP1 was abolished after treatment with CB (Fig. 10a, b). The expression levels of Osterix and VEGF-A also reduced significantly after treatment with CB (Fig. 10c, d, e). Treatment with BIO did not recover the nuclear translocation of YAP1 and β-Catenin under stimulation with CSFP (Fig. 10a, b). The expression levels of Osterix and VEGF-A also did not recover with the treatment of BIO (Fig. 10c–e**)**. These results showed that disruption of F-actin polymerization inhibited nuclear translocation of YAP1 and β-Catenin, and thereby inhibiting CSFP-induced osteogenic and angiogenic potential.

## Discussion

Here we report that F-actin and YAP-1 function as the regulators of β-Catenin to promote osteogenic and angiogenic abilities of MSC and TEL. Through overexpression, knockdown, and inhibition studies, we found that CSFP promoted the expression and nuclear translocation of β-Catenin to promote osteogenic and angiogenic abilities of MSC, thereby the bone and blood vessel formation of TEL. YAP-1 knockdown inhibited the nuclear translocation of β-Catenin and its transcriptional activity. Finally, disruption of F-actin polymerization inhibited the nuclear translocation of YAP1 and downstream events, such as nuclear translocation of β-Catenin, osteogenesis, and angiogenesis. Therefore, CSFP could promote the bone formation of TEL by regulating the F-actin/YAP1/β-Catenin signaling pathway.

Previous studies^8–10^ have already revealed the effect of CSFP on the osteogenesis, vascularization, and remodeling of TEL during vertebral laminae reconstruction. CSFP functions as cyclic pulsation stress^21^, which consistently impacts TEL’s dural surface and regulates the activities of skeletal stem cells. Our study found that CSFP could promote the expression of osteogenic and angiogenic markers in rat MSC derived from Wharton’s Jelly. Osterix is an osteoblast-specific transcription factor that activates a repertoire of genes during the differentiation of preosteoblasts into mature osteoblasts and osteocytes^22^. After intervention with CSFP, the Osterix^+^ cells increased significantly, indicating that CSFP could direct the osteogenic differentiation of MSC. VEGF-A also increased in the MSC after intervention with CSFP. VEGF-A played a vital role in the angiogenesis of tissue-engineered bone by stimulating the proliferation, migration, and tube formation of endothelial cells^23,24^. The increased expression of VEGF-A in MSC indicated better angiogenic abilities of TEL^23,24^.

β-Catenin activity is required for osteoblast lineage differentiation, and elimination of β-Catenin results in the lack of formation of cortical bone and spongy bone^25,26^. Chen et al.^27^ also showed that mice expressing an activated form of β-Catenin in osteoblasts exhibit increased osteogenic differentiation and bone matrix deposition. We found that CSFP activated β-Catenin by increasing its expression and nuclear translocation; β-Catenin overexpression could enhance the CSFP-induced osteogenic differentiation and angiogenic abilities of MSC, whereas knockdown or inhibition of β-Catenin led to a decrease in the osteogenic and angiogenic abilities. Further, inhibition of β-Catenin led to delayed blood vessel and bone formation in the TEL, consistent with results of in vitro study. β-Catenin played a crucial role in the CSFP-induced bone and blood vessel formation.

β-Catenin itself cannot sense and respond to biomechanical signals^11,12^. Recent studies showed that YAP/TAZ acts as a nexus orchestrating the interplay between cellular mechanics and developmental signaling cascades, allowing for the realization of mechanotransduction^11,12^. This mechanism raises the possibility that mechanical regulation of β-Catenin may function through the modulation of YAP/TAZ. Here we depict YAP and TAZ as equivalent proteins, and this is based on their structural similarities and genetic redundancy in a host of experimental conditions and biological functions^12,28^. CSFP could increase the protein levels of YAP1, but could not increase its mRNA expression levels, indicating that CSFP regulates the activity of YAP1 mainly by regulating its cytoplasmic degradation but not by regulating its transcription^29^. YAP1 and β-Catenin showed coexpression and colocalization trends at both cell and tissue levels, and CSFP could also promote their nuclear translocation and binding with each other. The interaction between YAP/TAZ and β-Catenin has been well studied: cytoplasmic YAP/TAZ facilitates the formation of the destruction complex and β-Catenin degradation, while nuclear YAP/TAZ cooperated with β-Catenin to regulate downstream transcriptional activities^11,20,30,31^. The knockdown of YAP1 led to decreased expression and cytoplasmic retention of β-Catenin, and inhibition of GSK-3β activity by BIO could reverse this trend significantly. Thus, we believed that the knockdown of YAP1 decreased the protein levels of β-Catenin mainly by inhibiting its degradation, not by transcription. CSFP could also promote the binding of YAP1 and β-Catenin, indicating that YAP1 might also assist with the transcriptional activities of β-Catenin^30^. In addition, nuclear YAP/TAZ can also assist with Runx2-regulatory transcriptional activities^32,33^, explaining that treatment with BIO could only recover part of the osteogenic and angiogenic abilities.

YAP/TAZ activity is regulated by the conformation and tension of the F-actin cytoskeleton, and F-actin reorganization could enhance YAP/TAZ activity^12,34^. F-actin can sense the surrounding mechanical cues and responded by reorganization to balance the tensional state^34^. F-actin reorganization leads to the nuclear translocation of YAP/TAZ and the downstream transcriptional activities^12,34^. Disruption of F-actin polymerization by Cytochalasin B led to cytoplasmic retention of YAP1 and β-Catenin, inhibiting the osteogenic and angiogenic abilities of MSC. Treatment with BIO could not recover the CSFP-induced osteogenic and angiogenic abilities of MSC, indicating that CSFP-induced osteogenesis and angiogenesis relied on nuclear translocation of YAP1 and β-Catenin. Cytoplasmic β-Catenin has to translocate to the nucleus to take effect, and its cytoplasm-to-nucleus transport relied on the F-actin cytoskeleton.

In conclusion, CSFP could promote the blood vessel and bone formation of TEL through the regulation of the F-actin/YAP1/β-Catenin signaling pathway. These findings advance our understandings of the vascularization and ossification of TEL and provide new therapeutic approaches for spinal degeneration after spinal laminectomy.

## Methods

### Ethics statement

We have complied with all relevant ethical regulations for animal testing and research. All animal experiments were performed in the Animal Facility of East China Normal University and according to the protocol (Protocol number: 20150482A168) authorized by the Animal Care and Use Committee of Fudan University.

### Cell culture and transfection

Rat mesenchymal stromal cell derived from Wharton’s Jelly were obtained from the Otwo Biotech (Guangzhou, China). All cells were cultured in Dulbecco’s modified Eagle’s medium (DMEM; Hyclone, UT, USA) supplemented with 10% fetal bovine serum (FBS; Biological Industries, Beit HaEmek, Israel).

Adenoviral constructs containing full-length *β-CATENIN* (Han-Bio, Shanghai, China) were used to generate stable *β-CATENIN* overexpressing cell lines. In all, 1 × 10^5^ MSC cells were plated in a 24-well plate and infected with *β-CATENIN* adenovirus at a multiplicity of infection of 100 in the presence of Lipofectamine 2000 (Invitrogen, CA, USA, 2 μl). After 24 h of incubation with adenovirus, cells were harvested for the determination of mRNA or protein expression levels of *β-CATENIN*.

For the establishment of transient *β-CATENIN*-knockdown or *YAP1*-knockdown cells, MSC was transfected with siRNAs (Ribo-Bio, Guangzhou, China). The sequences of siRNAs were listed as follows: si-*β-CATENIN*, 5′-GCACCATGCAGAATACAAA-3′; si-*YAP1*, 5′-GGTCAGAGATACTTCTTAA-3′. In all, 1 × 10^5^ MSC cells were plated in a 24-well plate and infected with siRNAs (50 nM) in the presence of Lipofectamine 2000 (2 μl). After 24 h of incubation, cells were harvested for the determination of mRNA or protein expression levels of *β-CATENIN* or *YAP1*.

### Inhibitors or agonists preparation

PD98059, XAV-939, wortmannin, 6-bromoindirubin-3′-oxime (BIO), and Cytochalasin B were purchased from MedChemExpress (NJ, USA). The chemical reagents were dissolved in dimethyl sulfoxide at a concentration of 10 mM, 10 mM, 1 mM, 10 mM, and 10 mM, respectively. And the final concentration of the chemical reagents in the culture medium was: 20 μM, 1 μM, 25 nM, 2 μM, and 20 μM, respectively.

### Bioreactor setting

To construct an in vitro cyclic pulsation mechanical model mimicking rat lumbar CSFP, the bioreactor setting parameters were set as below: flow rate at 15 cm/s, solenoid valve frequency at 300 times/min. MSC at passage 4–7 was evenly pipetted on sterile [poly(lactic-co-glycolic acid), PLGA] membranes (Nuoqi, Chongqing, China) to construct MSC-PLGA complexes. The MSC-PLGA complexes were divided into the cerebrospinal fluid pulsation (CSFP) group and cerebrospinal fluid pulsation isolation (Non-CSFP) group. In the CSFP group, the MSC-PLGA complexes were fixed onto the pulsation tube by the clip and connected to the bioreactor; while in the Non-CSFP group, the MSC-PLGA complexes were also fixed onto the pulsation tube by the clip and not connected to the bioreactor.

### Reverse-transcription PCR

Total RNA was isolated from cells or bone tissue using TRIzol Reagent (Invitrogen) according to manufacturer’s instructions. cDNA was synthesized from total RNA (500 ng) using reverse transcription kit (Takara, Tokyo, Japan). qPCR was performed in triplicate using 1 µl of cDNA in a standard SYBR premix Ex Taq (Takara) on the Applied Biosystems 7500 Real-Time PCR Detection System (Applied Biosystems, CA, USA). GAPDH served as an internal control. The following primers were used: *GAPDH*, 5′-GGCACAGTCAAGGCTGAGAATG-3′, 5′-ATGGTGGTGAAGACGCCAGTA-3′; *C-MYC*, 5′-GGTGGAAAACCCGACAGTCA-3′, 5′-TAGCGACCGCAACATAGGAC-3′; *CYCLIN-D1*, 5′-ACCAATCTCCTCAACGACCG-3′, 5′-CTCCTCGCAGACCTCTAGCA-3′*; β-CATENIN*, 5′-CCAAGTGGGTGGCATAGAGG-3′, 5′-CAGGCTCGGTAATGTCCTCC-3′; *YAP1*, 5′-CTTGACCCTCGTTTTGCCATGAA-3′, 5′-GACGGTCTGACATTTTGGAGCAT-3′; *RUNX2*, 5′-ATGGTTAATCTCTGCAGGTCACT-3′, 5′-CTGCTTGCAGCCTTAAATGA-3′; *OSTERIX*, 5′-TGAGGAAGAAGCCCATTCAC-3′, 5′-ACTTCTCCCGGGTGTG-3′; *BMP2*, 5′-CAGCGAGTTTGAGTTGAGG-3′, 5′-CGGTACAGGTCGAGCATAT-3′; *OCN*, 5′-CCTAGCAGACACCATGAGGA-3′, 5′-GTCAGAGAGGCAGAATGCAG-3′; *ALP*, 5′-GCACAACATCAAGGACATCG-3′, 5′-TCAGTTCTGTTCTTGGGGTACA-3′; *VEGF-A*, 5′-CGAACGTACTTGCAGATGTGAC-3′, 5′-GACCCAAAGTGCTCCTCGAA-3′; *FGF2*, 5′-GCGACCCACACGTCAAACTA-3′, 5′-ACTGGAGTATTTCCGTGACCG-3′; *MMP9*, 5′-ACGTCTTTCACTACCAAGACAAG-3′, 5′-GCAGGAGGTCATAGGTCACG-3′.

### EdU assay

Cell proliferation was measured with an EdU assay kit (Ribo-Bio, Guangzhou, China). Experiments were performed according to the manufacturer’s instructions. The representative images were obtained using a Leica fluorescence microscope.

### Red phalloidin staining

Cytoskeleton of MSC was stained with Acti-stain 555 Fluorescent Phalloidin (Cytoskeleton, CO, USA, 1:500) according to the manufacturer’s instructions.

### Immunofluorescence

Immunofluorescence was performed as previously described^10^. The following antibodies were used: β-Catenin mouse monoclonal (1:100), YAP1 mouse monoclonal (1:100), Runx2 mouse monoclonal (1:200), Osterix mouse monoclonal (1:500), VEGF-A mouse monoclonal (1:100), mouse IgG_1_-FITC isotype control (1:200, Santa Cruz, CA, USA), β-Catenin rabbit polyclonal (1:100), CD31 rabbit monoclonal (1:500, Severicebio, Wuhan, China), Rabbit IgG monoclonal isotype control (1:200, Abcam, MA, USA), Alexa Fluor^®^ 647 conjugated Goat Anti-Mouse IgG (1:100), Alexa Fluor^®^ 488 conjugated Goat Anti-Mouse IgG (1:100, Abcam, MA, USA), Cy5 conjugated Goat Anti-rabbit IgG (1:100), Alexa Fluor^®^ 488-conjugated Goat Anti-Rabbit IgG (1:100), Cy3 conjugated Goat Anti-Rabbit IgG (1:100, Severicebio), and DAPI (Leagene, Beijing, China).

### Immunohistochemistry

Immunohistochemistry was performed as previously described^10^. The following antibodies were used: BMP2 rabbit polyclonal (1:500), OCN rabbit polyclonal (1:200), VEGF-A rabbit monoclonal (1:300), and horseradish peroxidase (HRP)-conjugated secondary antibodies (Severicebio), mouse IgG_1_-FITC isotype control (1:200, Santa Cruz), Rabbit IgG monoclonal isotype control (1:200, Abcam).

### Co-immunoprecipitation

Cells were lysed in IP buffer (Servicebio) containing protease inhibitor cocktail (Servicebio). Total lysates (200 μg) were incubated with primary antibodies (1 µg) and Protein A/G agarose beads (Millipore, MA, USA) overnight at 4 °C with gentle shaking. The immunoprecipitated complexes were then washed with lysis buffer three times and eluted from the beads with protein loading buffer. The immunoprecipitated complexes were immunoblotted and subjected to mass spectrometry analysis (ClinX, ChemiScope 6300, MA, USA). Images were analyzed using ImageJ software.

### Western blotting analysis

Western blot was performed as previously described^35^. Total protein was extracted from the lysed samples in RIPA buffer with PMSF (Beyotime, Shanghai, China) on ice. Then cells were collected and centrifuged for 15 min (12,000 rpm, 4 °C). BCA Protein Assay kit was used to quantify protein concentration according to the manufacturer’s instructions (Thermo, MA, USA). The supernatants (20 μg protein) were subjected to SDS-PAGE gel and transferred onto PVDF membranes (Millipore). The nonspecific sites were blocked with 5% non-fat dried milk for 2 h at room temperature. The membranes were then incubated with primary antibodies for β-Catenin (Santa Cruz, 1:1000), YAP1 (Santa Cruz, 1:1000), and β-Actin (Santa Cruz, 1:1000) at 4 °C overnight. The membranes were incubated with goat anti-mouse IgG-HRP secondary antibody (Santa Cruz) at room temperature for 2 h. Finally, the ECL Detection kit (Beyotime, Shanghai, China) and Fluor Chem E system (Proteinsinple, CA, USA) were used for detection and photography. Images were analyzed by ImageJ software. All blots were derived from the same experiment and processed in parallel. Uncropped western blotting images were provided in Supplementary Fig. 4, where the size markers were labeled.

### Construction of tissue-engineered laminae

The hydroxyapatite-collagen I scaffold was bought from the Beijing Allgens Medical Science & Technology Co., Ltd. Under the sterile condition, the hydroxyapatite-collagen I scaffold was cut to the size of 8 mm × 6 mm. Rat MSC at passage 4–7 was trypsinized and resuspended in media at a concentration of 1 × 10^6^/ml. Then, 100 μl cell suspensions were pipetted on one side of each scaffold, and after 30 min, 100 μl cell suspensions were pipetted on the other side. All constructs were placed in culture medium before implantation.

### Live/Dead staining

Cell viability of MSC in TEL was measured with a Live/Dead assay kit (Best-Bio, Shanghai, China). Experiments were performed according to the manufacturer’s instructions. The representative images were obtained using a ZEISS confocal fluorescence microscope (ZEISS, Jena, Germany).

### Scanning electron microscope

The TEL were fixed with electron microscopy fixative solution (Severicebio), dehydrated, mounted on an aluminum stub, and sputter-coated with gold-palladium for 30 s. The morphology of TEL and MSC adhesion on the scaffolds was then viewed on a scanning electron microscope (Hitachi, Tokyo, Japan).

### Animal studies

The construction of CSFP and Non-CSFP were performed as previously described^8–10^. Briefly, for the CSFP animal model, spinous processes and interspinous ligaments were removed to expose the vertebral laminae, a bone defect measuring 8 mm × 6 mm × 1 mm was created in the vertebral laminae, leaving two fresh cancellous bone end measuring 8 mm × 1 mm × 2 mm, then the TEL were placed and fixed in the bone defect. For the Non-CSFP animal model, spinous processes and interspinous ligaments were removed to expose the vertebral laminae, a cancellous bone end measuring 8 mm × 2 mm was created in the outer cortex of laminae while preserving the dura surface cortex of laminae, similar to that of the CSFP group, then the TEL were fixed onto the native laminae. For the CSFP + XAV animal model, the surgical procedure was the same as the CSFP animal model; but after the procedure, the rats were injected intraperitoneally with XAV-939 according to the standard of 4 mg/kg at the frequency of twice a week for the first two weeks and once a week. (Supplementary Fig. 2).

### Statistical analysis

Statistical analysis was conducted using GraphPad Prism version 6.02 software program for Windows (GraphPad, CA, USA). All data are presented as mean ± SEM. One-way ANOVA or two-way ANOVA were used for analysis, and Tukey’s multiple comparisons test was used for comparison between groups. All tests were two-sided, and *P*-values < 0.05 were considered to be statistically significant.

### Reporting summary

Further information on research design is available in the Nature Research Reporting Summary linked to this article.

## Supplementary information

Supplementary information.

Reporting summary.

## Data Availability

All relevant data that support the findings of this study are available within this published article or available from the corresponding author upon reasonable request.
