# Supplementary material for: Cyclic pulsation stress promotes bone formation of tissue engineered laminae through the F-actin/YAP-1/β-Catenin signaling axis
Source: NPJ Regen Med. 2021 Sep 6;6:51. doi: 10.1038/s41536-021-00164-w (PMC8421434; doi:10.1038/s41536-021-00164-w)
Supplement: Supplementary file 1 — Supplementary information. [file 41536_2021_164_MOESM1_ESM.pdf]

Supplementary Information

Cyclic Pulsation Stress Promotes Bone Formation of Tissue Engineered Laminae through the F-actin/YAP-1/β-Catenin Signaling Axis

Linli Li[1#]; Hailong Li[1#]; Yiqun He[1]; Han Tang[1]; Jian Dong[1]; Xujun Chen[1]; Feizhou Lyu[1,2*]; Youhai Dong[1*]

1. Department of Orthopedics, Shanghai Fifth People's Hospital, Fudan University.

2. Department of Orthopedics, Huashan Hospital, Fudan University.

# Linli Li and Hailong Li contributed equally to this article.

*Correspondence to: Professor Youhai Dong, Email address: youhaidong1964@163.com; and Professor Feizhou Lyu, Email address: lufeizhou@hotmail.com. Address: No.128 Ruili Road, Minhang District, Shanghai, 200240, People's Republic of China.

Supplementary Figure 1

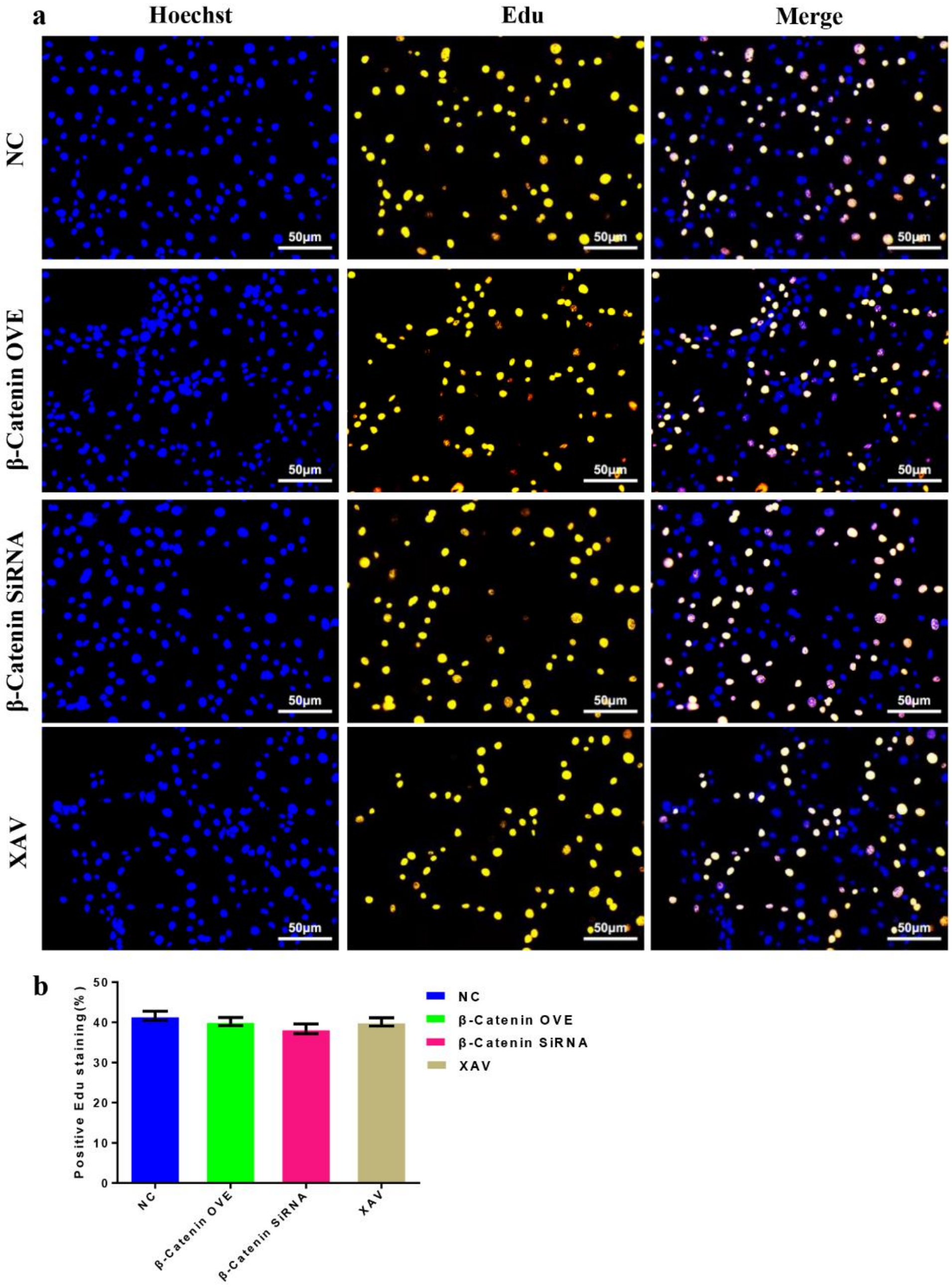

Supplementary Figure 1 CSFP did not affect the proliferative abilities of MSC through the regulation of β-Catenin. **(a)** Edu staining of the normal control (NC), β-Catenin-overexpression (β-Catenin OVE), β-Catenin-knockdown (β-Catenin SiRNA), and β-Catenin-inhibition (XAV) cell models; **(b)** There was no statistical difference about the percentage of MSC in proliferating phase between four groups. $P > 0.05$.

Supplementary Figure 2

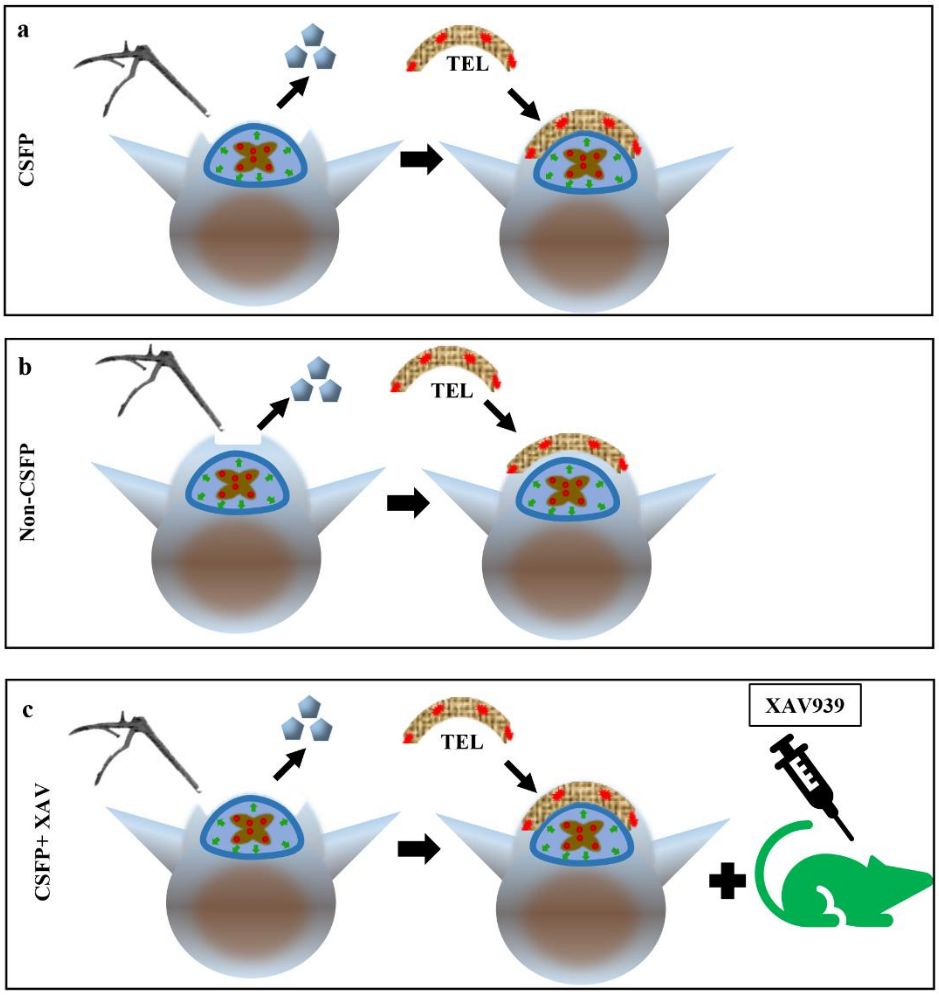

Supplementary Figure 2 Illustration diagram of CSFP rat models (CSFP group), CSFP isolation rat models (Non-CSFP group), and CSFP+ β-Catenin inhibition rat models (CSFP+XAV group). **(a)** For CSFP animal model, a bone defect measuring 8mm×6mm×1mm was created in the vertebral laminae, leaving two fresh cancellous bone end totally measuring 8mm×1mm×2, then the tissue-engineered laminae (TEL)was placed and fixed in the bone defect; **(b)** For the Non-CSFP group, a cancellous bone end measuring 8mm×2mm was created in the outer cortex of laminae while preserving the dura surface cortex of laminae, similar to that of CSFP group, then the TEL was fixed onto the native laminae; **(c)** For the CSFP+XAV group, the surgical procedure was the same with the CSFP animal model; but after the procedure, the rats were injected intraperitoneally with XAV-939 according to the standard of 4 mg/kg at the frequency of twice a week for the first two weeks and once a week.

Supplementary Figure 3

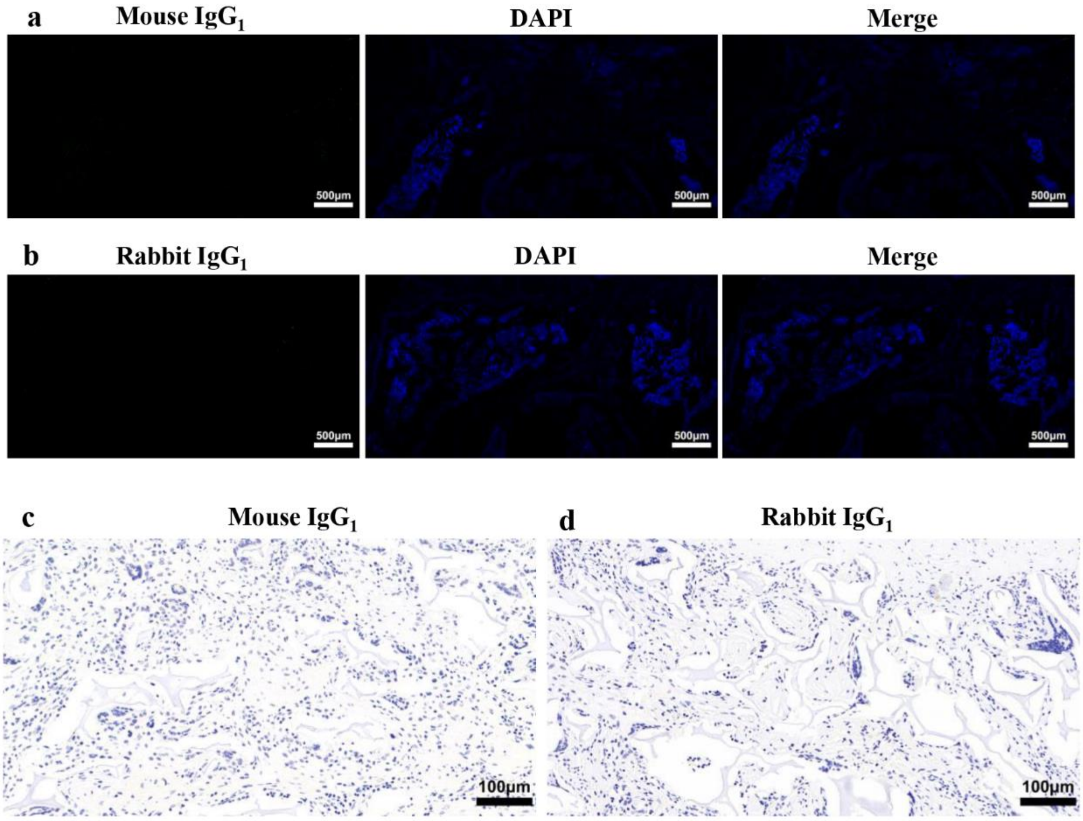

Supplementary Figure 3 Isotype controls for IF and IHC staining. **(a)** IF staining of mouse IgG_1 showed negative staining; **(b)** IF staining of rabbit IgG_1 showed negative staining; **(c)** IHC staining of mouse IgG_1 showed negative staining; **(d)** IHC staining of rabbit IgG_1 showed negative staining.

Supplementary Figure 4

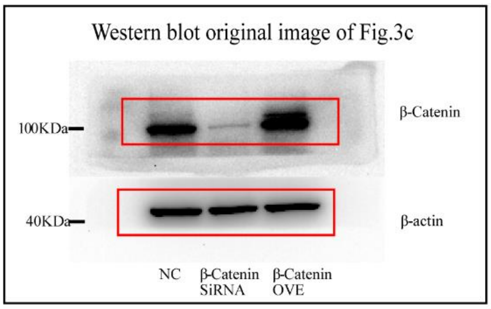

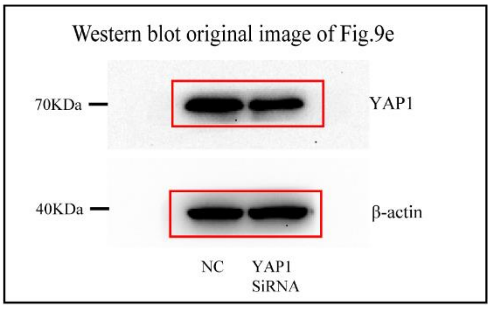

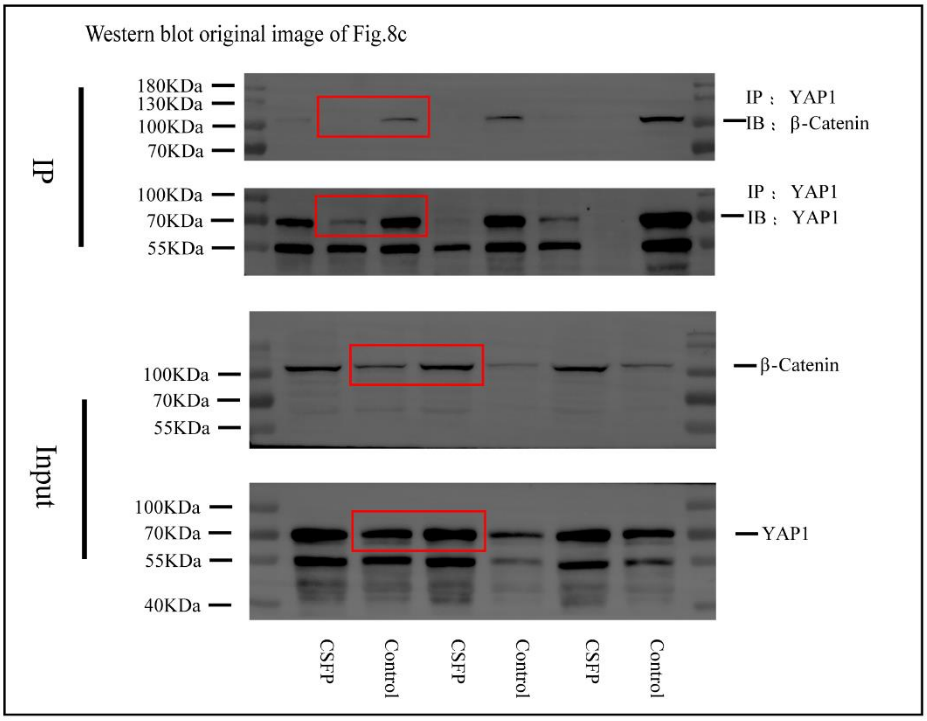

Supplementary Figure 4: Uncropped images of Western blots. Red boxes indicate areas that are cropped.
