# Supplementary material for: Cyclic pulsation stress promotes bone formation of tissue engineered laminae through the F-actin/YAP-1/β-Catenin signaling axis
Source: NPJ Regen Med. 2021 Sep 6;6:51. doi: 10.1038/s41536-021-00164-w (PMC8421434; doi:10.1038/s41536-021-00164-w)
Supplement: Supplementary file 2 — Reporting summary. [file 41536_2021_164_MOESM2_ESM.pdf]

nature portfolio

Corresponding author(s): Feizhou Lyu and Youhai Dong

Last updated by author(s): Aug 4, 2021

Reporting Summary

Nature Portfolio wishes to improve the reproducibility of the work that we publish. This form provides structure for consistency and transparency in reporting. For further information on Nature Portfolio policies, see our Editorial Policies and the Editorial Policy Checklist.

Statistics

For all statistical analyses, confirm that the following items are present in the figure legend, table legend, main text, or Methods section.

n/a	Confirmed	
☐	☒	The exact sample size (n) for each experimental group/condition, given as a discrete number and unit of measurement
☐	☒	A statement on whether measurements were taken from distinct samples or whether the same sample was measured repeatedly
☐	☒	The statistical test(s) used AND whether they are one- or two-sided<br>*Only common tests should be described solely by name; describe more complex techniques in the Methods section.*
☒	☐	A description of all covariates tested
☐	☒	A description of any assumptions or corrections, such as tests of normality and adjustment for multiple comparisons
☐	☒	A full description of the statistical parameters including central tendency (e.g. means) or other basic estimates (e.g. regression coefficient) AND variation (e.g. standard deviation) or associated estimates of uncertainty (e.g. confidence intervals)
☐	☒	For null hypothesis testing, the test statistic (e.g. F, t, r) with confidence intervals, effect sizes, degrees of freedom and P value noted<br>*Give P values as exact values whenever suitable.*
☒	☐	For Bayesian analysis, information on the choice of priors and Markov chain Monte Carlo settings
☒	☐	For hierarchical and complex designs, identification of the appropriate level for tests and full reporting of outcomes
☒	☐	Estimates of effect sizes (e.g. Cohen's d, Pearson's r), indicating how they were calculated

Our web collection on statistics for biologists contains articles on many of the points above.

Software and code

Policy information about availability of computer code

Data collection	GraphPad Prism 6.0 (GraphPad Software, Inc. San Diego, CA, United States)
Data analysis	GraphPad Prism 6.0 (GraphPad Software, Inc. San Diego, CA, United States)

For manuscripts utilizing custom algorithms or software that are central to the research but not yet described in published literature, software must be made available to editors and reviewers. We strongly encourage code deposition in a community repository (e.g. GitHub). See the Nature Portfolio guidelines for submitting code & software for further information.

Data

Policy information about availability of data

All manuscripts must include a data availability statement. This statement should provide the following information, where applicable:

- Accession codes, unique identifiers, or web links for publicly available datasets
- A description of any restrictions on data availability
- For clinical datasets or third party data, please ensure that the statement adheres to our policy

The data generated in this study are available from the corresponding author on reasonable request.

Field-specific reporting

Please select the one below that is the best fit for your research. If you are not sure, read the appropriate sections before making your selection.

☒ Life sciences ☐ Behavioural & social sciences ☐ Ecological, evolutionary & environmental sciences

For a reference copy of the document with all sections, see nature.com/documents/nr-reporting-summary-flat.pdf

Life sciences study design

All studies must disclose on these points even when the disclosure is negative.

Sample size	No sample size calculations were performed. Three or more biological replicates were carried out for each experiment.
Data exclusions	No exclusions
Replication	Replicates for experiments were successful and are reflected within the results.
Randomization	For in vivo studies all animals were randomly allocated into each group.
Blinding	Images were assigned randomized file names for the assessor.

Reporting for specific materials, systems and methods

We require information from authors about some types of materials, experimental systems and methods used in many studies. Here, indicate whether each material, system or method listed is relevant to your study. If you are not sure if a list item applies to your research, read the appropriate section before selecting a response.

Materials & experimental systems

n/a	Involved in the study	
☐	☒	Antibodies
☐	☒	Eukaryotic cell lines
☒	☐	Palaeontology and archaeology
☐	☒	Animals and other organisms
☒	☐	Human research participants
☒	☐	Clinical data
☒	☐	Dual use research of concern

Methods

n/a	Involved in the study	
☒	☐	ChIP-seq
☒	☐	Flow cytometry
☒	☐	MRI-based neuroimaging

Antibodies

Antibodies used	β-Catenin mouse monoclonal (Santa Cruz, sc-7963), YAP1 mouse monoclonal (Santa Cruz, sc-101199), Runx2 mouse monoclonal (Santa Cruz, sc-101145), Osterix mouse monoclonal (Santa Cruz, sc-393060), VEGF-A mouse monoclonal (Santa Cruz, sc-7269), mouse IgG1-FITC isotype control (Santa Cruz, sc-2855), β-Catenin rabbit ploypclonal (Severicebio, GB11015), CD31 rabbit monoclonal (Severicebio, GB12063), BMP2 rabbit polyclonal (Severicebio, GB11252), OCN rabbit ploypclonal (Severicebio, GB11233), VEGF-A rabbit monoclonal (Severicebio, GB13034), Rabbit IgG monoclonal isotype control (Abcam, ab172730), Goat Anti-Mouse IgG Alexa Fluor® 647 (Abcam, ab150115), Goat Anti-Mouse IgG Alexa Fluor® 488 (Abcam, ab150113), Cy5 conjugated Goat Anti-rabbit IgG (Severicebio, GB21303), Alexa Fluor® 488-conjugated Goat Anti-Rabbit IgG(Severicebio, GB21303), Cy3 conjugated Goat Anti-Rabbit IgG (Severicebio, GB21303), and horseradish peroxidase (HRP)-conjugated secondary antibodies (Severicebio, G1215).
Validation	β-Catenin mouse monoclonal (Santa Cruz, sc-7963, https://www.scbt.com/p/beta-catenin-antibody-e-5?requestFrom=search), YAP1 mouse monoclonal (Santa Cruz, sc-101199, https://www.scbt.com/p/yap-antibody-63-7?requestFrom=search), Runx2 mouse monoclonal (Santa Cruz, sc-101145, https://www.scbt.com/p/runx2-antibody-27-k?requestFrom=search), Osterix mouse monoclonal (Santa Cruz, sc-393060, https://www.scbt.com/p/osx-antibody-e-6?requestFrom=search), VEGF-A mouse monoclonal (Santa Cruz, sc-7269, https://www.scbt.com/p/vegf-antibody-c-1?requestFrom=search), mouse IgG1-FITC isotype control (Santa Cruz, sc-2855, https://www.scbt.com/p/normal-mouse-igg1-fitc?requestFrom=search), β-Catenin rabbit polyclonal (Severicebio, GB11015, https://www.servicebio.cn/goodsdetail?id=1320), CD31 rabbit monoclonal (Severicebio, GB12063, https://www.servicebio.cn/goodsdetail?id=593), BMP2 rabbit polyclonal (Severicebio, GB11252, https://www.servicebio.cn/goodsdetail?id=1476), OCN rabbit polyclonal (Severicebio, GB11233, https://www.servicebio.cn/goodsdetail?id=1458), VEGF-A rabbit monoclonal (Severicebio, GB13034, https://www.servicebio.cn/goodsdetail?id=4296), Rabbit IgG monoclonal isotype control (Abcam, ab172730, https://www.abcam.com/rabbit-igg-monoclonal-epr25a-isotype-control-ab172730.html), Goat Anti-Mouse IgG Alexa Fluor® 647 (Abcam, ab150115, https://www.abcam.com/goat-mouse-igg-hl-alexa-fluor-647-ab150115.html), Goat Anti-Mouse IgG Alexa Fluor® 488 (Abcam, ab150113, https://www.abcam.com/goat-mouse-igg-hl-alexa-fluor-488-ab150113.html), Cy5 conjugated Goat Anti-rabbit IgG (Severicebio, GB21303, https://www.servicebio.cn/goodsdetail?id=4191), Alexa Fluor® 488-conjugated Goat Anti-Rabbit IgG(Severicebio, GB21303, https://www.servicebio.cn/goodsdetail?id=273), Cy3 conjugated Goat Anti-Rabbit IgG(Severicebio, GB21303, https://

www.servicebio.cn/goodsdetail?id=253), and horseradish peroxidase (HRP)-conjugated secondary antibodies (Severicebio, G1215, https://www.servicebio.cn/goodsdetail?id=1894).

Eukaryotic cell lines

Policy information about cell lines

Cell line source(s)	Rat mesenchymal stromal cell derived from Wharton's Jelly (Otwo Biotech, Guangzhou, China)
Authentication	The cell line was not authenticated
Mycoplasma contamination	The cell line was tested for mycoplasma by the supplier and sent to us mycoplasma free.
Commonly misidentified lines (See ICLAC register)	n/a

Animals and other organisms

Policy information about studies involving animals; ARRIVE guidelines recommended for reporting animal research

Laboratory animals	Adult (8 weeks, male, Sprague-Dawley) rats were used in this study.
Wild animals	The study did not involve wild animals
Field-collected samples	The study did not involve field collected samples
Ethics oversight	All animal protocols were approved by the Institutional Animal Care and Use Committee of Fudan University.

Note that full information on the approval of the study protocol must also be provided in the manuscript.
